# Antileishmanial potentials of azacitidine and along with meglumine antimoniate on *Leishmania major*: *In silico* prediction and *in vitro* analysis

**DOI:** 10.1371/journal.pone.0291321

**Published:** 2023-09-08

**Authors:** Ali Derakhshani, Iraj Sharifi, Ehsan Salarkia, Alireza Keyhani, Setareh Agha Kuchak Afshari, Behzad Iranmanesh, Mahdieh Lashkarizadeh, Hamid Nejad Biglari, Moslem Lari Najafi, Mehdi Bamorovat

**Affiliations:** 1 Research Center for Hydatid Disease in Iran, Kerman University of Medical Sciences, Kerman, Iran; 2 Leishmaniasis Research Center, Kerman University of Medical Sciences, Kerman, Iran; 3 Medical Mycology and Bacteriology Research Center, Kerman University of Medical Sciences, Kerman, Iran; 4 Department of Dermatology, Afzalipour Hospital, Afzalipour Faculty of Medicine, Kerman University of Medical Sciences, Kerman, Iran; 5 Department of Pathology and Stem Cell Research Center, Kerman University of Medical Sciences, Kerman, Iran; 6 Department of Surgery, Faculty of Medicine, Kerman University of Medical Sciences, Kerman, Iran; 7 Pharmaceutical Sciences and Cosmetic Products Research Center, Kerman University of Medical Sciences, Kerman, Iran; Iran University of Medical Sciences, ISLAMIC REPUBLIC OF IRAN

## Abstract

This study aimed to investigate the *in vitro* and *in silico* antileishmanial activity of azacitidine (AZA) on *Leishmania major* promastigotes and amastigotes. The *in silico* method was used to evaluate the possibility of the interaction of AZA into the binding pocket of inducible nitric oxide synthase (iNOS), a leading defensive oxidative metabolite. Following that, *in vitro* anti-promastigote, and anti-amastigote activity of AZA was determined using an MTT assay and a macrophage model, respectively. Cytotoxic effects of AZA and meglumine antimoniate (MA) were also assessed by MTT assay on murine macrophages. All experiments were performed in triplicate. The results showed that AZA interacted with Ser133, Gln134, and Lys13 amino acids of iNOS, and the molecular docking score was obtained at -241.053 kcal/mol. AZA in combination with MA significantly (P<0.001) inhibited the growth rate of nonclinical promastigote (IC_50_ 247.6±7.3 μM) and 8.5-fold higher of clinical intramacrophage amastigote stage (29.8±5.3 μM), compared to the untreated group. A significant upsurge of Th1 subsets and transcription genes and a meaningful decline in Th2 cytokines subclasses at the equivalent concentrations of AZA and MA was observed (P<0.001). The apoptosis effect of AZA along with MA was significantly induced on *L*. *major* in a dose-dependent manner (P<0.001). The present study demonstrated that AZA possesses antileishmanial activity in *in vitro* and *in silico* models. However, AZA combined with MA was more effective than AZA alone in inhibiting the growth rate of promastigotes and amastigotes of *L*. *major*. This study indicates that AZA in combination with MA demonstrated a potent antileishmanial mechanism, promoting immune response and enhancing an immunomodulatory role toward the Th1 pathway. This experimental study is a basic study for applying more knowledge about the mechanisms of AZA along with MA in animal models in the future.

## Introduction

Leishmaniasis is a human-animal protozoan disease prevalent in most parts of the world in approximately 98 countries [1]. Currently, about 12 million people are infected with various types of leishmaniasis (visceral and cutaneous), and at the same time, it is estimated that about 1 billion people are exposed to it [1–3]. In total, 90% of leishmaniasis occurs in 7 countries, including Afghanistan, Iran, Algeria, Peru, Brazil, Saudi Arabia, and Syria [4].

Antimony compounds such as meglumine antimoniate (MA, Glucantime®) have been the basis of leishmaniasis treatment since 1911 and are considered the first line of treatment for this disease [5]. The use of these compounds has limitations such as the long course of treatment, the high cost of drugs, the lack of response to treatment in about 10%-15% of cases, and severe toxicity to the heart, liver, and kidneys [6–9].

On the other hand, the use of second-choice compounds including amphotericin B, paromomycin, pentamidine, terbinafine, and other byproducts have faced similar limitations of costs, high dosages, long parenteral duration, poor efficacy profile, and the emergence of parasite resistance [9–12]. Over time, various methods have been used to treat cutaneous leishmaniasis (CL) such as topical radiotherapy, lesion burning, cryotherapy, and local infiltration, which are not very useful [13]. Therefore, it seems necessary and vital to study drugs that have no previous problems.

Azacitidine (AZA) is a chemical analog of cytidine that is used as a chemotherapeutic agent for myelodysplastic syndrome, myeloid leukemia, and cancers. AZA can act as an inhibitor of DNA methyltransferase direct cytotoxicity on abnormal cells [14, 15]. Some investigations have been conducted on the effectiveness of this drug in preventing infection by human immunodeficiency and T-lymphotropic viruses [16]. A study demonstrated that AZA has an early effect on the expression of genes associated with T lymphocytes and a delayed effect on the genes methylation activity. Also, it may influence the allogeneic transplantation setting as an immunomodulatory medicine [17]. Epigenetic regulation of cytokine genes including IL-2, IFN-γ, and IL-4 is a major approach in the initiation response of the immune system, and as mentioned AZA may play a role in the gene expression of T lymphocytes [17–19].

This study aimed to assess leishmanicidal potentials and action mechanisms of AZA, MA, and in combination including ligand-protein molecular docking, anti-leishmanial effect, safety index, immunomodulatory potential, and apoptotic profile against *Leishmania major* stages.

## Material and methods

### In silico assay

#### Estimation of practical residues of iNOS protein

To detect the hot spot of valuable residues in the construction of inducible nitric oxide synthase (iNOS) considering the importance of controlling *Leishmania* parasites before tying up, the “Hotspot” (https://loschmidtchemi.muni.cz/hotspotwizard/) and CASTp (http://sts.bioe.uic.edu/castp/index.html?1ycs) software were used [20, 21].

#### Study of physical pockets of iNOS protein

Outlining and measuring open superficial areas on 3-dimensional (3-D) constructions are essential in advanced experimentations. Accordingly, Molegro Virtual Docker software (Molegro 2011) was utilized to detect pockets on surfaces and cavities.

### Protein-ligand docking

The 3-D structure of azacitidine was achieved by PubChem CID 936 [PubChem https:// https://pubchem.ncbi.nlm.nih.gov/compound/Azacitidine]. The 3-D form of iNOS was obtained from the Protein Data Bank (PDB) (https://https://doi.org/10.2210/pdb1hig/pdb), and molecular docking investigations were completed in Molegro ApS (Aarhus 2.5.0, Denmark).

### In vitro study

#### Drug preparation

This work was undertaken as a case-control study. AZA (Sigma-Aldrich, St Louis, MO, USA) was obtained from commercial sources in Iran. Meglumine antimoniate (MA, Glucantime, Sanofi-Aventis, Paris, France) was considered the positive control drug and combinatory. Serial dilutions were prepared to obtain concentrations of 12.5, 25, 50, 100, and 200 μg/ml.

#### Parasite culture

*L*. *major* promastigotes from standard strain (MHOM/IR/Mash2) were maintained from Kerman Leishmaniasis Research Center and were cultured in RPMI-1640 medium (Biosera, France) enriched by 10% of fetal bovine serum (Sigma, USA) along with the penicillin-streptomycin antibiotic and incubated at 25±1˚C.

#### Macrophage culture

Murine macrophage cell-line (J774-A1) was maintained from Pasteur Institute (Tehran, Iran) and cultivated in DMEM medium (Sigma, USA) that was enriched with 10 % FBS (Sigma, USA) and penicillin together 0.5% streptomycin (Biosera, France). Cells were kept at 37°C and 5 % CO_2_.

#### Anti-promastigote activity

The activity of AZA, MA, and a combination of them against *L*. *major* promastigotes were determined by MTT assay. For this purpose, 10^5^ per ml of logarithmic phase promastigotes were counted and cultured in 96 well-plates and treated with several concentrations (12.5, 25, 50, 100, and 200 μg/ml) of drugs and then incubated at 25±1˚C for 72 h. Next, 10 μL of MTT solution was added to each well and kept for 3h after that 100 μL of DMSO was added to each well and the OD absorbance was read at 490 nm by Microplate Spectrophotometer BioTek EPOCH Elisa reader. Finally, the 50% inhibitory concentration (IC_50_) rate was determined using the SPSS package.

#### Anti-amastigote activity

10^5^ per ml of J774-A1 cells were counted and cultured on a glass microscope slide and incubated for 24 h (37°C with 5% of CO_2_). Next, 10^6^ per ml stationary phase promastigotes were added to macrophages (in ratio 10:1) and incubated for 24 h to let the parasites change to amastigote form. After that several concentrations (12.5, 25, 50, 100, and 200 μg/ml) of AZA, MA, and co-administration were added and incubated for 72 h. The samples were dried and fixed with methanol and next stained with Wright-Giemsa. The number of intramacrophage amastigotes was counted in 100 macrophages for each concentration and the mean of them was used for calculating IC_50_ values of amastigotes.

#### Cytotoxic effects of AZA and MA

For the evaluation of cytotoxicity on the J774-A1 cells, various concentrations (12.5, 25, 50, 100 and 200 μg/ml) of AZA, MA, and co-administration of them were added to the cells and incubated for 72h incubated cells (37°C, 5% CO2) in 96 well plates. 10 μl of MTT solution was added to each well and kept for 3 h. After that 100 μl of dimethyl sulfoxide (DMSO) was added to wells and optical density (OD) values were read by Bio-Tek ELISA-reader on 490 nm. Test performed triplicate for each concentration and finally compare with the untreated control group.

#### Quantitative real-time PCR

qPCR assay was used to define the relative expression of IFN-γ, IL-12, TNF-α, and iNOS in treated and untreated control groups. At first, the whole RNA of samples was extracted by using the total RNA Extraction Kit (Pars tous-Iran) according to the relevant protocol. Next, the quality and concentrations (ng) of RNA were measured by a Thermo Fisher NanoDrop device. The cDNA was made utilizing the BioRad cDNA synthesis kit (US). Finally, the qPCR process was carried out by the Rotor-Gene Q device (Corbett Research Rotor-Gene 3000, Australia) using the YTA SYBR Green qPCR MasterMix (Yekta Tajhiz, Iran). Table 1 shows the primer-template and control gene sequences. Gene expression was assessed using 2^-ΔΔCT^ techniques, and the CT was computed as follows: [ΔCT = CT (target)–CT (control)].

**Table 1:** The specific primers and reference gene sequences.

Template	Forward and reverse sequences (5´-3´)		Product size (bp)
**IFN-γ**	Forward	5-GCCGATGATCTCTCTCAAGTGAT-3	106
	Reverse	5-ACAGCAAGGCGAAAAAGGATG-3	
**IL-12**	Forward	TGGTTTGCCATCGTTTTGCTG	171
	Reverse	ACAGGTGAGGTTCACTGTTTCT	
**TNF-α**	Forward	CAGGCGGTGCCTATGTCTC	161
	Reverse	CGATCACCCCGAAGTTCAGTAG	
**iNOS**	Forward	ACATCGACCCGTCCACAGTAT	89
	Reverse	CAGAGGGGTAGGCTTGTCTC	
**GAPDH**	Forward	5-AGGTCGGTGTGAACGGATTTG-3	95
	Reverse	5-GGGGTCGTTGATGGCAACA-3	

#### Flow cytometry analysis

The apoptosis inducer of treated L. major promastigotes was determined by flow cytometry assay. Apoptosis Detection Kit (eBioscience, USA) was applied as instructed by the manufacturer. For that 10^6^ *L*. *major* promastigotes in the logarithmic phase were treated with several concentrations of AZA, MA, and co-administration and incubated for 72 h. PBS and binding buffer (1X) use to wash and prepare the samples. V-FITC (5 μl) and PI stain (5 μl) were added and incubated at ambient temperature for 20 min. in the dark. Apoptosis was determined by flow cytometry (BD FACSCalibur, USA).

### Statistical analyses

SPSS ver. 22.00 (Chicago, IL, USA) and GraphPad Prism ver. 8.0 (CA, USA) was used to define the difference between groups by using a paired T-test and one-way ANOVA. The significant cut-off for the P*-* value was < 0.05.

## Results

### Molecular docking

**Fig 1:**
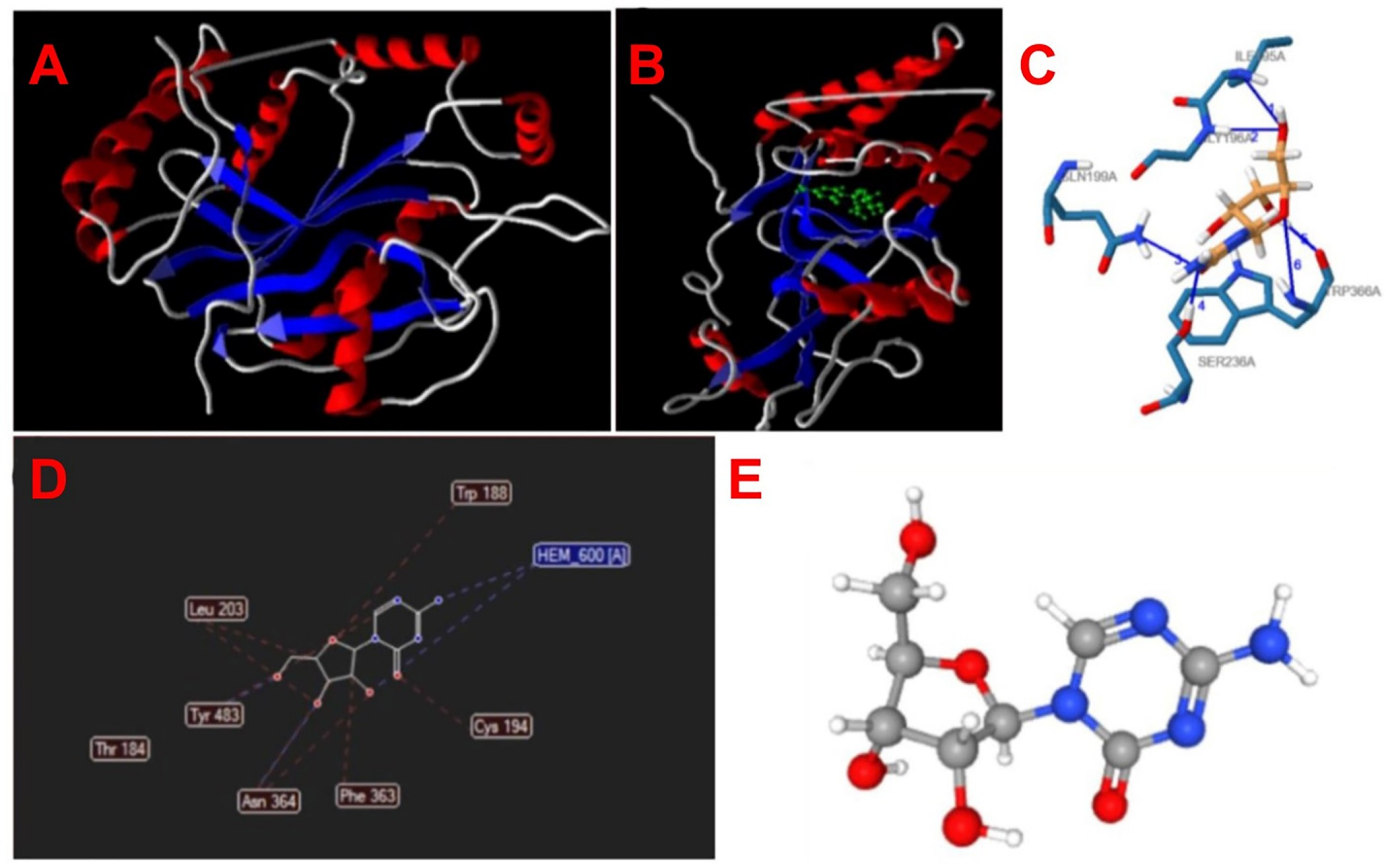
A) Nitric oxide, B) AZA binds to NO with the active site residues by LIGPLOT program, C) Predicted amino acids in pocket formation by PLIP web tool, D) Molecular docking by Molgro Virtual Docker software, E) 3D structure of AZA.

For molecular docking of competence of AZA for binding to iNOS, Thr184, Leu203, Thy483, Asn364, Phe363, Cys194, and Trp188 were predicted as suitable amino acids in mutable residues in catalytic pockets and access tunnels. Also, the structural and functional activity of the iNOS protein surface is presented in Fig 1A. The evaluated 2-D interaction diagrams show hydrophobic interaction and hydrogen bond binding energy of the ligand and target protein. A ligand interaction profiler (PLIP) online server was used to evaluate the interaction between iNOS and AZA metabolites (Fig 1B). Regarding steric relations, AZA interacts with Thr, Leu, Thy, Asn, Phe, Cys, and Trp amino acids of iNOS (Fig 1C). The results show that the MolDock score was 11.4238 kcal/mol with 17 heavy atoms (Fig 1D). Table 2 showed the ligand atom energies and contribution of the iNOS residues/molecules with AZA.

**Table 2:** Ligand atom energies and contribution of the iNOS residues/molecules with azacitidine.

ID		Name			Total		EPair			EIntra	
0		O			3.03		0			0.674325	
1		O			-2.34307		0			-1.6984	
2		O			3.59176		0			4.47732	
3		O			1.53647		0			2.83756	
4		O			-1.19951		0			0.670142	
5		N			-0.74477		0			0.293911	
6		N			1.13419		0			0.282154	
7		N			1.8059		0			1.98069	
8		N			-1.3937		0			1.14683	
9		C			-1.01585		0			0.10913	
10		C			-1.64143		0			0.838876	
11		C			-0.06996		0			1.12617	
12		C			0.212086		0			1.10703	
13		C			-0.29015		0			1.07419	
14		C			-1.39345		0			-0.69395	
15		C			0.694143		0			1.59185	
16		C			1.02739		0			1.5856	
**Hydrogen bond**											
**Index**	**Residue**		**AA**	**Distance H-A**		**Distance D-A**		**Donor angle**	**Donor atom**		**Acceptor atom**
1	195A		ILE	2.40		3.11		129.12	805 [Nam]		2936 [O3]
2	195A		GLY	2.55		3.23		125.97	814 [Nam]		2936 [O3]
3	199A		GLN	2.45		3.21		134.41	853 [Nam]		2937 [O2]
4	236A		SER	1.82		2.78		176.92	1236 [O3]		2939 [Nam]
5	366A		TRP	1.98		2.96		178.20	2935 [O3]		2344 [O2]
6	366A		TRP	2.98		3.75		134.58	2341 [Nam]		2933 [O3]

### In vitro findings

#### Effect of AZA, MA, or in combination on *L*. *major* promastigotes

**Fig 2:**
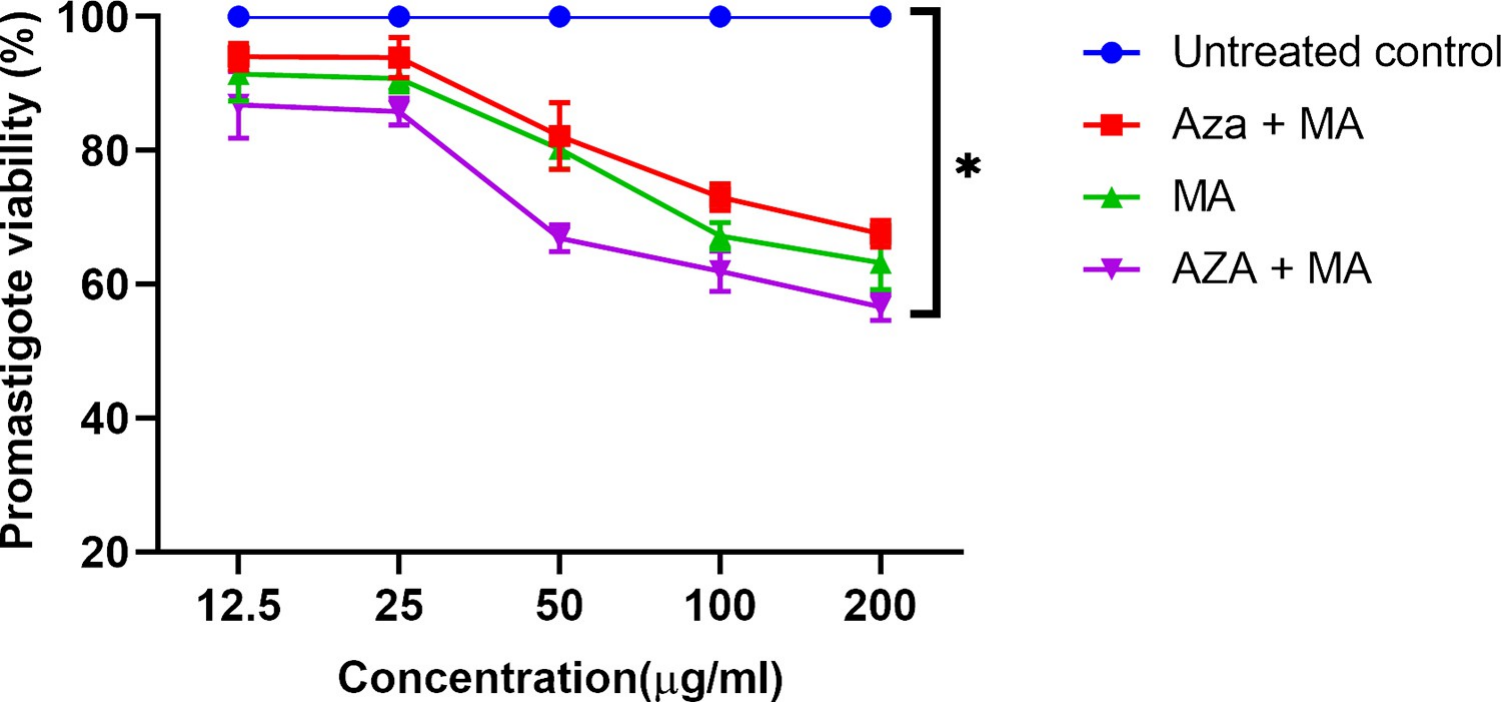
Viability rate of *L*. *major* promastigotes at different azacitidine and MA concentrations, alone or combined, relative to untreated control by MTT assay (*P<0.001).

The total average viability values of *L*. *major* promastigotes treated with different doses of AZA, MA, and combination are accessible in Fig 2. The results showed that the average viability of *L*. *major* promastigotes significantly decreased by increasing the concentration of AZA, MA, or a combination of them, which confirmed the anti-leishmanial activity of these drugs (P<0.001).

#### Effect of AZA and MA on *L*. *major* intra-macrophage amastigotes load

Indices of *L*. *major* intra-macrophage amastigotes differed significantly from the untreated group (P<0.001). IC_50_, CC_50,_ and SI of AZA, MA, and in combination are presented in Table 3 at the anticipated concentrations of each drug, cytotoxicity examination revealed no lethal effect as the SI was within the safety range (SI = CC_50_/IC_50_ ≥1).

**Table 3:** Evaluation of the IC_50_ values of azacitidine and meglumine antimoniate against amastigotes and promastigotes forms of *Leishmania*. *major*, compared to meglumine antimoniate and CC_50_ values of the drugs on macrophages using the SI index.

Drugs	Amastigote		Promastigote		Macrophage	^c^SI
	^a^IC_50_ ± SD (μg/mL)	P-value	^a^IC_50_ ± SD (μg/mL)	P-value	^b^CC_50_ (μg/mL)	(Selectivity index)
**MA**	52.3±8.4	NR	311.5±61	NR	590.9	11.3
**AZA**	103.2±24	P<0.001	413.7±28	P<0.001	1939.2	10.6
**AZA + MA**	29.8±5.3	P<0.001	247.6±7.3	P<0.001	479.7	16.1

^an^ IC_50_: Drug concentration that inhibited 50% of growth in promastigotes and amastigotes

^b^ CC_50_: Drug concentration that inhibited 50% of growth in macrophages

^c^ SI: Selectivity index (CC_50_/IC_50_)

AZA: Azatadine

NR: Not related

The number of intracellular amastigotes was significantly reduced at various medication doses (P<0.001), compared to the untreated control group (Table 4).

**Table 4:** The effect of different concentrations of azacitidine and meglumine antimoniate on the mean number of intra-macrophage amastigotes.

Concentration (μg/mL)	AZA		MA		AZA+MA	
	Mean±SD	P value	Mean±SD	P value	Mean±SD	P value
**0.0 (Control)**	41.35±1.91	NR	41.35±1.91	NR	41.35±1.91	NR
**6.25**	38.80±0.28	P>0.05	30.65±2.90	P<0.001	25.14±2.46	P<0.001
**12.5**	32.20±0.28	P<0.01	26.20±4.95	P<0.001	22.45±1.06	P<0.001
**25**	30.25±1.77	P<0.001	25.95±1.91	P<0.001	19.95±0.21	P<0.001
**50**	24.15±3.04	P<0.001	24.50±2.55	P<0.001	16.60±1.41	P<0.001
**100**	22.67±2.35	P<0.001	15.68±0.40	P<0.001	9.25±2.33	P<0.001
**200**	13.13±2.65	P<0.001	6.75±1.20	P<0.001	0.00±0.00	P<0.001

#### Effect of AZA, MA, and in combination on T-lymphocytes related gene expression profile

**Fig 3:**
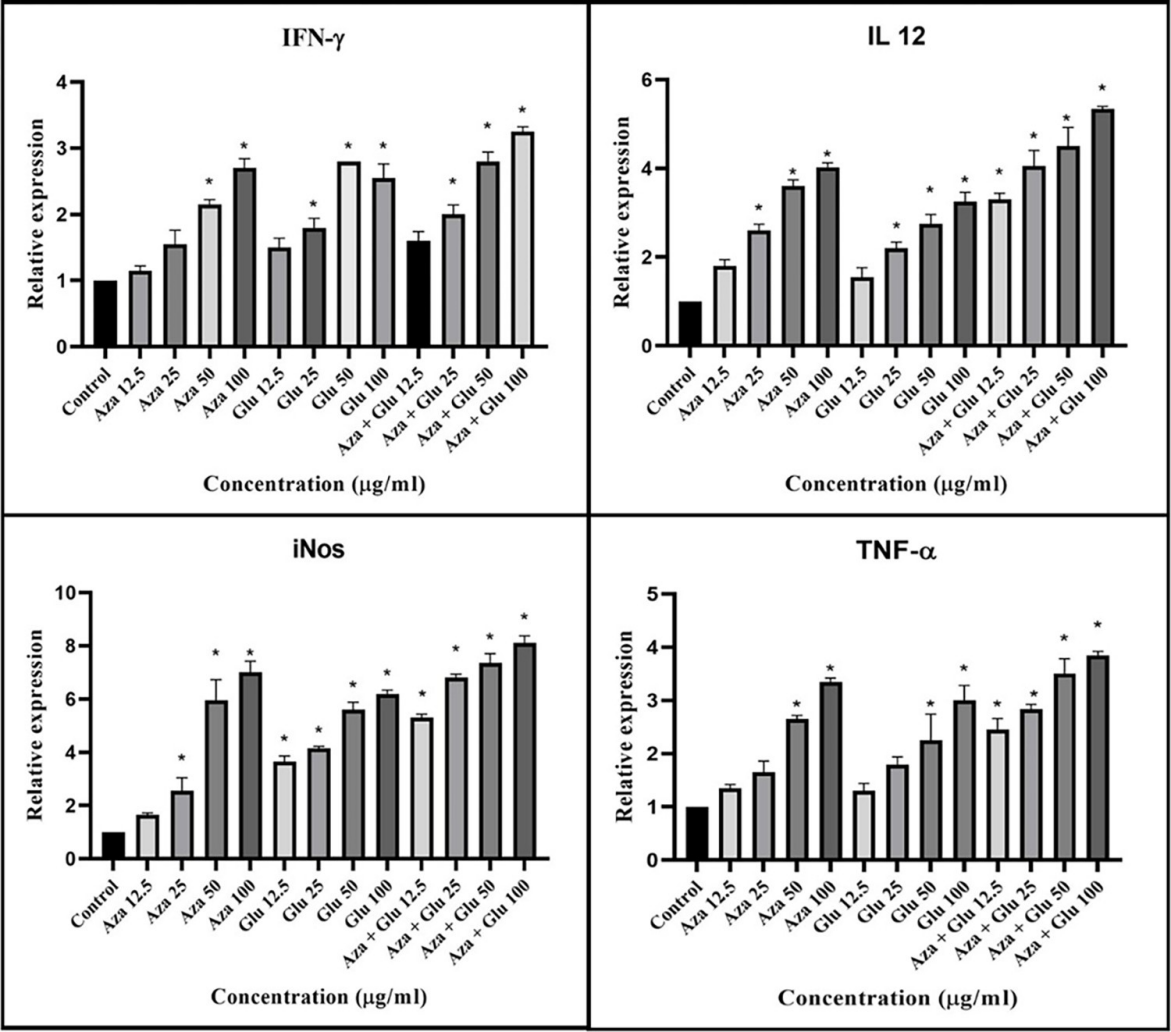
Th1 cytokines expression profile of IFN-γ (A), IL-12p40 (B), iNOS (C), and TNF-α (D) in Mφs treated with different concentrations of azacitidine, MA, and both, compared to the untreated group. Error bars are SD (**P<0.01, ***P<0.001, and ****P<0.0001). Each test was performed in triplicate.

Comparison of relative changes (the mean 2^-ΔΔCT^) of expression of cytokines (IFN-γ, IL-12, TNF-α, and iNOS) displayed elevated levels in the treated macrophages. The gene expression profiles in Th1 and Th2 phenotypes and transcription factors in the AZA and MA alone were the same. However, a significant upsurge of Th1 subsets and transcription genes and a meaningful decline in Th2 cytokines subclasses at equivalent concentrations were observed (P<0.001) (Fig 3).

#### Effect of AZA, MA, and both on apoptosis in *L*. *major*

**Fig 4:**
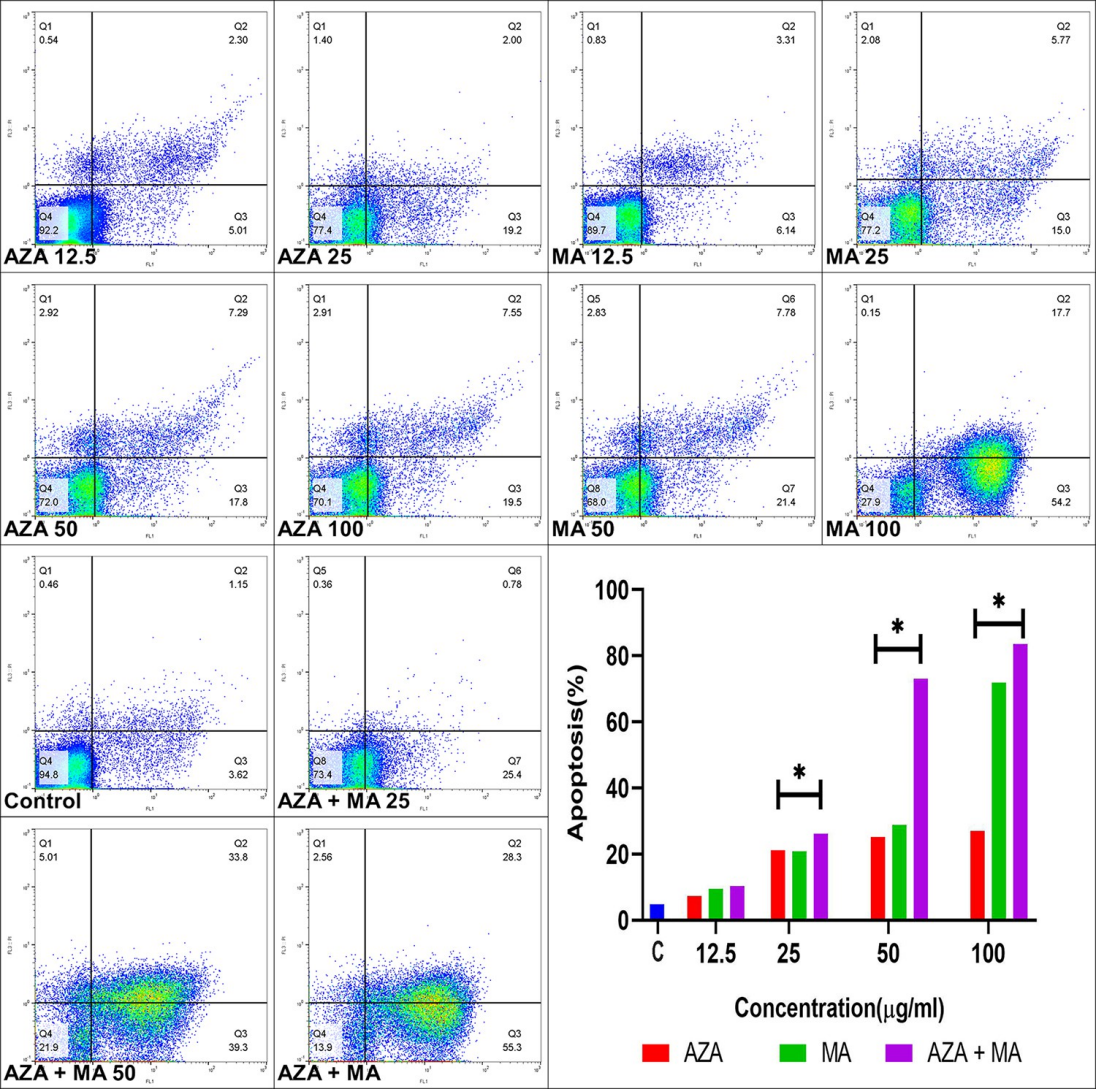
Flow cytometric analysis of *Leishmania major* promastigotes treated with different concentrations of azacitidine, MA, and in combination (difference was compared to untreated control (*P<0.001).

The effect of different concentrations of AZA, MA, or in combination on the apoptosis profile of *L*. *major* promastigotes show that the percent of apoptosis significantly increased in 25, 50, and 100 μg/ml of all drugs compare untreated control group (P<0.001). The results show in 12.5 μg/ml of AZA, MA, or combination, there was no effect (Fig 4).

## Discussion

Leishmaniasis is a serious public health problem, and chemical drugs to treat it have many side effects and parasite resistance [22, 23]. Extensive efforts to develop an effective and affordable vaccine have been unsuccessful [24, 25]. The control of so many vectors and hosts is not feasible [26]. Therefore, the mainstay of management strategy is based on chemotherapeutic agents [27]. The present challenges require a more efficient treatment modality and the improvement of novel drugs alone or in combination for leishmaniasis treatment. Based on the previous findings, various treatment modalities have been applied for CL with various consequences [11, 28–30]. Previous studies have confirmed the effects of cytidine nucleotide analogs with known inhibitory activity against DNA methyltransferase (DNAMT). AZA is a ring analog of cytosine that differs from the natural nucleoside by having nitrogen in place of carbon in the pyrimidine’s position five. This drug is known as a competent drug to display multiple pharmacological activities such as anti-cancer, anti-parasitic, anti-inflammatory, and anti-microbial effects. This factor is effective in preventing cancer cell survival; therefore, it has been suggested as a therapeutic agent for the treatment of acute myelogenous leukemia. It has also been found that AZA is located in DNA and inhibits the methylation pattern of specific regions of the gene while activating related genes. AZA-induced epigenetic changes in hepatocytes result in significant improvements in metabolic and enzymatic activity, compared to untreated cells [31–33].

Uridine-cytidine kinase converts AZA to triphosphate, which is then degraded by cytidine deaminase. AZA is a type of ribonucleoside that binds to RNA and a less extent DNA. It inhibits DNA methyltransferase (DNAMT1), the enzyme responsible for methylating DNA, in a non-competitive manner after incorporation into DNA. In addition to generating DNA methylation, DNAMT1 has two domains that can attract histone deacetylase (HDAC1 and HDAC2). Blocking histone deacetylation with drugs could further reduce DNAMT1 activity. Inhibition of DNAMT1 by AZA is not observed in resting cells and occurs at AZA concentrations that do not cause significant DNA synthesis suppression [34–36]. A study displayed that AZA inhibits schistosome egg production and anti-fecundity activity with increased DNA methylation in the schistosome parasite (*Schistosoma mansoni*) [37]. The findings of another study showed the effects of DNA methylation in the *Entamoeba histolytica* parasites that were treated with AZA, a potent inhibitor of DNA methyltransferase. Drug treatment modulated the expression of amebic genes by approximately 2.1% [38]. Also, the results of a study demonstrated the effect of AZA on *Streptococcus pneumoniae*, specifically on biofilm formation, and the expressions of genes involved. AZA inhibits in vitro biofilm formation and reduces the expression genes involved in the synthesis (autoinducer-2 as by-products of the methionine recycling pathway) [39].

Before experimentations, we predicted the preferred orientation of the ligand-protein molecular docking, and signal transduction of AZA to iNOS, a lethal and key oxidative metabolite generated by active macrophages against the leishmanial agents [10]. These molecules were tightly bound together to form a stable complex. The binding affinity helped us in predicting the strength of association, the main reason for conducting this investigation. Documentation of the binding behavior plays a fundamental role in the rational design of drugs, cost-effectiveness, time-saving, and clarifying biochemical processes [40].

*In silico* study show, the antileishmanial activity of AZA was demonstrated via different aspects. Our study showed that AZA interacted with Thr, Leu, Thy, Asn, Phe, Cys, and Trp amino acids of iNOS and binds to it and reduces NO release via reducing the activity of iNOS.

Previous studies showed AZA in combination with Venetoclax reduced the expression of multiple amino acid transporters, especially on cysteine over-expressions in leukemia stem cells (LSC) [41]. At several AZA concentrations, we saw a significant increase in Th1 subsets and transcription genes, as well as a significant reduction in Th2 cytokines subclasses. Also, the apoptosis effect of AZA was dose-dependently induced on *L*. *major*. In previous reports, preclinical studies involving human promyelocytic leukemia HL-60 cells treated with varying concentrations of AZA suggested that the mechanism of apoptosis differed depending on the drug concentration [42]. In past studies, the effects of AZA on the process of cell apoptosis have been reported mostly through the caspase cascade activation mechanism, and mitochondrial morphological changes and damage to this organelle were not visibly observed. One of the most effective things in this process is methylation changes in the cell. This also leads to the continuation of the apoptosis process and its induction to the nearby cells even despite not receiving the drug [42–44].

Maybe low concentrations of AZA mainly resulted in drug incorporation into RNA and cytotoxicity in G1 cells and higher concentrations were linked to changes in DNA and RNA metabolism, resulting in cell death in the G1 and S phases+. In cell-line models, AZA also showed differentiation-inducing activity at low concentrations and strong antileukemic effects at high concentrations. As a result, AZA’s efficacy as an anticancer agent appears to be due to two distinct mechanisms: cytotoxicity (high dose) and hypomethylation induction, resulting in cellular effects other than immediate cytotoxicity (low dose). More research was done to see how AZA affected cytokines that regulate hematopoiesis, such as LIF, oncostatin M, IL-6, and IL-11. Using cell culture supernatants obtained from healthy and rheumatoid arthritis patients’ peripheral blood mononuclear cells, AZA caused downregulation of these cytokines in rheumatoid arthritis patients’ mononuclear cells but not in healthy subjects. As previously reported [41, 45] AZA treatment resulted in an increase in IFN+ NK cells, CD4+ T cells, and CD8+ T cells [46, 47].

The infection of macrophages with *L*. *major* primmed upregulation of iNOS, an important molecule to combat the *Leishmania* parasite, although the mode of actions and the significances of these counteractions on the intracellular human parasites are mainly unknown. However, nitric oxide (NO) is an important antipathogenic effector and cellular signaling molecule. Intracellular amastigotes can takeover macrophages as a microenvironment for replication. In addition, its conventional role as an anti-microbial mechanism [47], which is produced by iNOS, offers a critical immunomodulatory response. Phagocytic cells not only establish the focal infected cellular partition but also express the iNOS in response to T lymphocyte-derived interferon-gamma (IFN-Ɣ).

To provide compelling evidence for selecting drug activity we surveyed and monitored a vast number of opposing immunomodulatory genes variably expressed between different treatment groups. The immunostimulatory mode of the action exerted by AZA, particularly when combined with SbV involved with CD4+ T helper1(Th1) inherently plays a crucial role in the self-controlling of leishmaniasis more profoundly in the presence of proper treatment modality. Th1 phenotypes secrete cytokine activators of cell-mediated immunity (CMI), including IFN-Ɣ, IL-12, and TNF-α. On the other hand, the expression of Th2-related cytokines (IL-4, IL-10, and TGF-β) was depressed as they are associated with humoral immunity and promoting antibody response [7, 28].

Furthermore, the selectivity index was calculated to evaluate the drug toxicity value. Various drug concentrations alone or in combination showed no cytotoxic effect when mammalian cells were treated as characterized by an SI ≥ 1, non-toxic [48]. The safety level was calculated from the ratio between CC_50_ for murine macrophages and the IC_50_ for intracellular amastigotes.

AZA demonstrated a powerful activity on both stages of *L*. *major* but the effect was more significantly profound on amastigotes, especially when used in combination with the conventional drug as evidenced by the low IC_50_ value. A similar outcome was observed when *in vitro* natural components were used against *L*. *major* amastigotes. AZA exhibited a significantly less pronounced effect on extracellular promastigotes than intra-macrophage amastigotes. The susceptibility of the clinical stage to AZA relative to promastigotes is mainly due to the reduced level of drugs by amastigotes. These two stages are biochemically and physiologically different in response to respiratory stress mediators and this difference has principally been the basis of leishmaniasis chemotherapy [49]. Further research is necessary to understand the mechanisms by which the combination of AZA and MA work synergistically and to use the optimal dose for treatment.

## Conclusion

These results showed a remarkable leishmanicidal effect by AZA alone and greater when combined with MA. This combination demonstrated multiple synergistic mechanisms of action as signified by upregulation of Th1 and iNOS phenotypes, potent high safety index, and high apoptotic profile. Molecular docking performed by bioinformatic modeling which involved the interaction between AZA and iNOS, the main oxidative metabolite in leishmaniasis control was the source for predicting the strength of association and conducting this investigation. From our perspective, this study is unique because no comparable research has inclusively been conducted before. This experimental study is a basic study for applying more knowledge about the mechanisms of AZA along with MA in animal models in the future.

## Supporting information

S1 Checklist STROBE statement—checklist of items that should be included in reports of observational studies. (PDF)
